# The Book World of Medicine and Science

**Published:** 1895-07-20

**Authors:** 


### THE BOOK WORLD OF MEDICINE AND SCIENCE.

CASES OF SPORADIC CRETINISM TREATED BY THYROID EXTRACT. By TELFORD TELFORD-SMITH, M.A., M.D., Medical Superintendent, Royal Albert Asylum. (Lewes: South Counties Press.)

This is a reprint from "The Journal of Mental Science" for April, 1895, of a paper which was delivered at the last annual meeting of the British Medical Association. Four cases are recorded, and their improvement is shown by reproductions of photographs. Dr. Telford-Smith says in regard to the question of dosage that two conditions—the temperature at night and the state of general nutrition—afford a useful guide as to when sufficient of the thyroid extract is being administered. If the temperature can be kept at about 97·5 deg. to 98 deg. Fahr., and at the same time no emaciation is set up, the physiologically useful dose has been gauged; but if flesh is lost steadily, and any degree of emaciation is set up, the dose should be diminished, even though the temperature still remain low.

A MANUAL OF GYNÆCOLOGICAL PRACTICE FOR STUDENTS AND PRACTITIONERS. By Dr. A. DUHRSSEN, Privat Docent in Midwifery and Gynæcology in the University of Berlin. Translated by JOHN W. TAYLOR, F.R.C.S., Surgeon to the Birmingham and Midland Hospital for Women; and FREDERICK EDGE, M.D.Lond., M.R.C.P., F.R.C.S., Surgeon to the Wolverhampton and District Hospital for Women. (London: H. K. Lewis. 1895. pp. 241.)

For those who like to find a great deal of information in a small space this manual of modern practice will be very welcome. In the short space of 240 pages it not only gives a very complete sketch of the general subject, but enters into details regarding certain portions which are much more full than can be found in many much larger treatises. At the same time, it must be confessed that concentrated knowledge, especially when translated, does not form altogether easy reading. The chapters on gynæcological examination and on the disinfection of instruments, &c., are likely to be found very useful. It is to be noted that while sterilization by heat is advised for all instruments, dressings, and other appliances, including the operating coat of the surgeon, lysol is recommended for use upon the patient, and there can be no doubt that if the 1 per cent. solution of this substance should prove to be as efficacious as the perchloride of mercury and carbolic acid so generally employed, a great deal of trouble and much complication will be saved by using a single antiseptic for all purposes. A certain caution must be exercised in accepting this book as a guide in operative procedures, for while in many parts it represents generally accepted views, there are many points in regard to which the opinions expressed are purely personal. For example, the views put forward as to vaginal laparotomy and vaginal fixation of the retroflexed uterus will certainly not meet with universal acceptance, although it is a matter of very great interest to note the freedom with which it has been found possible to open the peritoneum and obtain access to all parts of the uterus by that route. On the other hand, it should be said that this work has the great advantage of being no compilation; on every page there are signs that it is the outcome of practical experience, the directions given are often exact and minute to a degree, and the illustrations, although in some cases rather crude, are thoroughly illustrative of the points involved. The foot-notes added by the translators are uniformly good and common sense, and add considerably to the value of the book.

YEAR-BOOK OF THE SCIENTIFIC AND LEARNED SOCIETIES OF GREAT BRITAIN AND IRELAND. Twelth Annual Issue. (London: Charles Griffin and Co. 1895.)

That the yearly publication of such a book as this should be a commercial possibility is a sign of the vitality of the learned societies and of the interest taken in their work. Comprising as it does lists of papers read during 1894 before societies engaged in fourteen departments of research, together with the names of their authors, its compilation must have been a work of considerable labour. The book contains a large amount of information which could be found in no other single work; its utility to scientific men would, however, have been much increased had it been found possible to arrange a subject index of the papers read.

THE GREAT EASTERN RAILWAY COMPANY'S TOURIST GUIDE TO THE CONTINENT. Edited by PERCY LINDLEY. Illustrated, and with maps. Price 6d. (London: 30, Fleet Street.)

A good handbook to those parts of the Continent accessible to the tourist by the Hook of Holland route has just been issued by the Great Eastern Railway Company, under the editorship of Mr. Percy Lindley. In addition to tours in Holland and Belgium (more fully discussed in the Editor's "Walks in Belgium"), more extended journeys to Germany and Switzerland are mapped out for the traveller, always presupposing a start from Harwich *via* the Hook. The guide does not profess to supply the names of hotels, or much practical information of the Baedeker stamp, about the various places mentioned, but the very full descriptions of points of interest will no doubt prove of good service in showing the traveller what he should see, and (even more important) why he should see it. The maps are excellent.

